# Supplementary material for: Learning curve and surgical outcome of robotic assisted colorectal surgery with ERAS program
Source: Sci Rep. 2022 Nov 29;12:20566. doi: 10.1038/s41598-022-24665-w (PMC9709162; doi:10.1038/s41598-022-24665-w)
Supplement: Supplementary file 1 — Supplementary Information 1. [file 41598_2022_24665_MOESM1_ESM.zip › LSC Raw data-Ñ[▒K/052/OP N_ñw╝╨░O▒Kñσ.pdf]

W95 / W95

臺中榮民總醫院
手術室護理記錄

[redacted]

基本資料

- 室別： 開刀房-06室，手術科別： CRS，手術類別： 常規，日期： 2019/10/18
- 進入時間： 1400 ； 麻醉開始時間： 1420 ； Time Out時間： 1505 ； 劃刀時間： 1508 ； 手術結束時間： 1810 送出時間： 1835 ；
- 麻醉方式： GE
  診斷： Malignant tumor of rectum
  手術名稱： 1.Low anterior resection with colo-anal anastomosis2.Ileostomy3.Laparoscopy4.
  手術局部用藥： nil.
  術前預防性抗生素：
  - 抗生素名稱一： Veterin 1GM IVD由麻醉護理師給予
  - 時間： 1450
  - 上次簽章: 張秀敏

  ，追加預防性抗生素：
- 手術醫師： 蔣鋒帆，江世偉，楊曙榮，林楙時
- 麻醉醫師： VS 沈靜慧
-
  - 刷手護士A ： 陳秋誼，時間： 1400，接替刷手一： 胡怡珮，時間： 1500，接替刷手二： 邱欐媃，時間： 1800
  - 流動護士A ： 張秀敏，時間： 1400，接替流動一： 高子貽，時間： 1500，接替流動二： 邱欐媃，時間： 1730
- 更多接替及流動護士： 接替流動三： 高子貽，時間： 1800

皮膚狀況

A-V Shunt： 無
術前： 完整
　記錄者： 上次簽章: 張秀敏
術後： 完整
　記錄者： 高子貽

手術準備

手術臥式 單· ·手術臥式

-
  - 手術臥式： 截石臥式
  - 使用附件： 溫毯 38 ℃，矽膠枕頭，腳架，其它： 頭架
  - 皮膚準備： 10%Tr.Betadine，10%Aq Betadine，其它： 2%chlorhexidine
  - 電燒： 使用電燒: 電極片位置： 其它： 電擊迴路墊，記錄者： 上次簽章: 張秀敏

止血帶T： 無

植入物

- 植入物名稱/號碼/數量/部位： ENDO GIA 60X4.1*3 for colon

，記錄者： 上次簽章: 高子貽

標本送檢

- 細菌培養 無
- 細胞學 無
- 冰凍切片 無
- 病理標本
  組織名稱一： rectum
  ,
  組織名稱二： distal cutend
  ,
  組織名稱三： IMA Root

- 截肢 無

標本交付 標本： 3 袋 ，標本： 0 盒 ，標本： 0 瓶 ，細胞學： 0 管 ，Culture Tube： 0 管 ，被交付醫師： R 林楙時，

- 記錄者： 上次簽章: 高子貽
- 時間： 1515

傷口術後數料

傷口數料：

- 請於圖上標示部位：

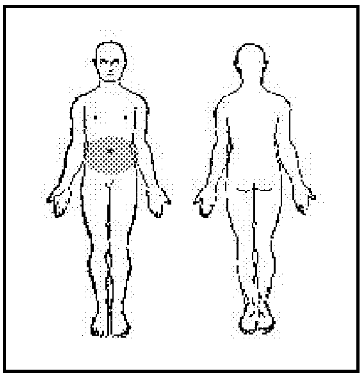

- 傷口數料： MENPORT

導管

導管	天數	導管資訊	插入日期	插入時間	更換日期	拔除日期	拔除時間	拔除原因	預計拔除/更換	預計拔除/更換日期
Foley(1)	1	號碼:16 Fr.; 部位:膀胱;	2019/10/18	1430						
NG Tube(1)	1	號碼:16 Fr.; 部位:胃; 註記:fix 60cm;	2019/10/18	1415						
Penrose Drain(1)	1	號碼:12 mm.; 部位:骨盆腔;	2019/10/18	1645						

用物計數

- 手術中出血量： minimai c.c.
- 手術中尿量： 50 c.c.
-
    - 其它： 無

用物計數： 單一切口

- 1.器械： 正確，2.縫針： 正確，3.刀片： 正確，4.紗布： 正確，5.腹部墊： 未使用，6.紗球： 未使用，7.PEANUTS ： 未使用，8.紗條： 未使用，9.絲帶： 正確，10.針頭： 未使用，11.Vessel loop： 未使用，12.Boots： 未使用，13.Jelco： 未使用，14.棉條： 未使用，
    - 第一次：
        - 刷手護士 ： 胡怡珮
        - 流動護士： 上次簽章: 高子貽
        - 時間： 1700

  ,
    - 第二次：
        - 刷手護士 ： 胡怡珮
        - 流動護士： 上次簽章: 高子貽
        - 時間： 1720

  ,
    - 第三次：
        - 刷手護士 ： 邱獼婇
        - 流動護士： 上次簽章: 高子貽
        - 時間： 1800

備註

備註： 腹腔鏡NO.4

- 記錄者： 上次簽章: 張秀敏

- 時間： 1500

填寫護理人員 高子貽

正式摘要

	W95 / W95

doctorRank:B
